# From 2D projections to the 3D rotation matrix: an attempt for finding a machine learning approach for the efficient evaluation of mechanical joining elements in X-ray computed tomography volume data

**DOI:** 10.1007/s42452-022-05220-8

**Published:** 2022-12-12

**Authors:** T. M. Schromm, C. U. Grosse

**Affiliations:** Chair of Non-Destructive Testing, Technical University of Munich, Franz Langinger Str. 10, 81245 Munich, Germany

**Keywords:** Non-destructive testing, X-ray, Computed tomography, Mechanical joining, Rivets, Machine learning, Rotation

## Abstract

Destructive and predominantly manual procedures are commonly used in the automotive industry for the testing of mechanical joints, such as rivets or screws. Combining X-ray computed tomography (CT) and machine learning (ML) bears the potential of a non-destructive and largely automated methodology. Assuming the desired result is a comprehensible and documentable evaluation, three basic steps need to be automatized: First, a joint must be detected and identified as such in a CT scan of the joined parts. Second, the detected region containing the joint is rotated to a predefined orientation. Third, key measures in cross-sections from the newly oriented joint are dimensioned and documented. This work deals only with the second step, the rotation. On the one hand, we present a methodology for creating a well-curated data set for the contextual machine learning application. On the other, we evaluate its performance on the well-known ResNet50. More concretely, we investigate if it is possible for a deep convolutional neural network (CNN) to learn the respective rotation matrix from three volume projections that are perpendicular to each other. Two scenarios are investigated: In one scenario we assume that future data that is presented to the network has similar rivet demographics to historic data. We therefore do not employ hold-out sets for the network evaluation. In the other scenario we assume the opposite and therefore evaluating the networks performance with hold-out sets. We show that from a machine learning point of view, a CNN like ResNet50 is well able to learn this relationship with acceptable accuracy. In most cases the validation loss dropped below 0.1 after only a couple of epochs. In one particular case, we even reached both mean and median errors lower than 0.2 for approximately 80% of the entire test set of 1600 examples using our methodology. From an application point of view, however, these low test set errors should be treated with caution since small deviations from the intended rotation matrix can cause volume warping and translation. In another case, in which we used a hold-out set, only a fraction of the median errors were below 0.2.

## Introduction

### Structural quality evaluation of processed rivets

**Fig. 1:**
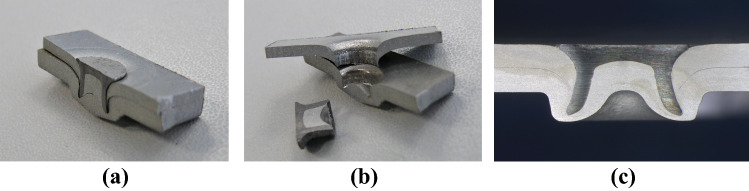
Cross-sectional views of two different rivets. **a** Cross-sectioned rivet (composed). **b** Cross-sectioned rivet (decomposed). **c** Close-up of a cross-sectioned rivet

The structural quality of mechanical joints, for example self-piercing rivets, is predominantly evaluated via macro-sectioning (see, for example, [1–3, 10]). This method, however, is of a destructive nature (Figs. 1a, b), provides only 2D information (Fig. 1c), and includes several steps that need to be performed in a manual fashion (see, for example, [13, 16]). More concretely, and in chronological order, macro-sectioning involves the following steps: (1) forcefully removing the mechanical joint with parts of its structural environment from a compound structure like a body in white, (2) carefully and steadily sawing it in half, 3) grinding and polishing the exposed surface, (4) treating it with chemicals,^1^ and eventually, (5) investigating the joint’s cross-section under a microscope. In the last step, key measures like undercut/interlock, head protrusion, or die-side material thickness are assessed. Throughout the entire workflow, care must be taken not to deform the joint by applying too much force. In addition to that, the described processes always depend to a certain extent on the skills of the person performing them. This introduces an unwanted subjectivity to the evaluation process.

The purpose of our work is to eliminate manual, destructive evaluations of mechanical joints in the automotive industry. In a larger endeavor, we therefore investigate the possibilities of a non-destructive approach to automatically evaluate the structural quality of mechanical joints, such as self-piercing rivets (e.g., Fig. 1). The endeavor’s objective is to realize a workflow that combines X-ray computed tomography, machine learning, and conventional image processing. Three basic steps need to be automatized for this: First, a joint must be detected and identified as such in a CT scan of the joined parts. This should be rather easy since it is basically only a binary classification task and there are lots of publications that demonstrate how well state-of-the-art classification networks perform on such tasks. See for example [7]. Here they reach over 90% top-1 accuracy on ImageNet [9]. Second, the detected region containing the joint is rotated to a predefined orientation. Third, key measures in cross-sections from the newly oriented joint are dimensioned and documented.

This work, however, focuses only on a specific subsection in that endeavor, namely on the automatic alignment of a CT volume that contains a randomly oriented rivet. Note that an automated and accurate alignment of the CT volume is necessary for an efficient and precise dimensioning of the joint.

### The potential and applications of X-ray CT and machine learning

Since the discovery of X-rays in the 19th century [15], and the development of computed tomography in the second half of the 20th century [4, 5] X-ray CT has since become an indispensable tool in medical applications. In addition to medicine, X-ray CT is now used frequently throughout other sectors such as in the food, automotive, aerospace as well as metrology and quality inspection industries (e.g., [5, 40, 41]).

The number of use cases for machine learning and its subdomains like deep learning have exploded since the early 2010s,^2^ and the fields of application have become increasingly diverse. To give only some examples besides computed tomography [6, 8], successful applications now range from finance and economics [20, 21, 25], medicine [26–28], sports [30, 31] to language [32, 33] music composition [34, 35] and the film industry [36, 37]. This shows the versatility and potential of machine learning algorithms.

In order to show that machine learning algorithms can and have been successfully applied to X-ray and CT data we mention some applications in the following: Lots of work was published on how deep learning can help to analyze chest X-rays [24]. Machine learning was also employed to generate synthetic ground truth data for pore detection in CT data of cast aluminium parts [23]. Another publication [14] shows that when applying deep learning algorithms to clinical data, including CT, of COVID-19 patients they reached an improved prognosis performance on the severity of the patients compared to other existing scores. In [29], they obtained high precision values on classifying three types of welding defects with a relatively small X-ray data set and data augmentation.

### Automated alignment of mechanical joints

Using X-ray CT means transitioning into 3D volumetric space, which in turn considerably increases the amount of available information about the structure of the sample and its condition. Gaining the same amount of information with destructive measures and reasonable processing times is not possible, even with above average proficiency in metallography. At this point of the argument, the 2D, destructive, and entirely manual evaluation process of mechanical joints, which was mentioned in Sect. 1.1, is replaced by a hypothetical process that provides 3D information and is of non-destructive nature. Yet, manually evaluating the information gained from 3D space still remains a necessity.

The manual evaluation of an X-ray CT volume that contains a mechanical joint would include region of interest (ROI) detection, aligning the volume for further processing and easier inspection, and, finally, classification and/or dimensioning of previously defined characteristics of the joint. These tasks represent clearly distinguishable processes, which means, in theory, automating them individually should be possible.

The physical samples and CT volumes utilized in this work have been processed for and utilized in our previous work [22]. The aim there was to automatically and non-destructively find, extract, and align mechanical joining elements from an X-ray computed tomography volume. We explored the robustness of a rather simple approach based on *Otsu* thresholding [43] and a principle component analysis (PCA). We found that the thresholding was negatively influenced by strong artifacts and that the PCA was relatively susceptible to minor geometric deviations in some cases. However, we also showed that our approach worked really well on sample geometries with clearly distinguishable eigenvectors (e.g., flow-drilling screws).

Classical image processing approaches are rule-based. Hence, one must be aware of most, if not all, eventualities in order to conceive a reliably performing algorithm. One would have to know and explicitly define a large number of rules that cover these eventualities. The principle of machine and deep learning, however, is that an algorithm derives abstract rules from a large number of examples. So instead of writing the rules, one must conceptualize an in silico structure that has enough mathematical potential to learn^3^ complex mathematical mappings or rules, respectively.

In this work, we investigate the possibility of an automated alignment of mechanical joints in an X-ray CT volume by means of convolutional neural networks. Furthermore, we investigate two scenarios. In one scenario, we assume that the demographic features of the rivets that are being processed and subsequently analyzed are constant and known. In the second scenario, we assume the opposite, meaning the process chain was subjected to changes^4^ leading to rivet demographics that the network has never seen before. We explain our approach in detail in the next section.

## Methods

**Fig. 2:**
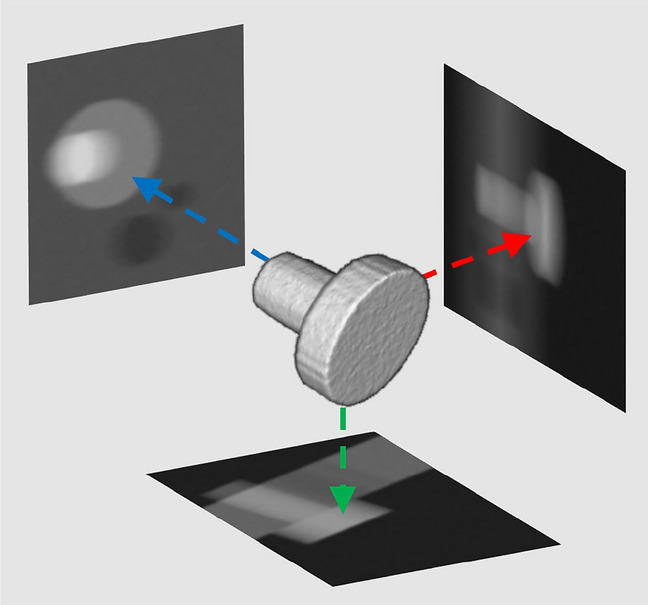
Perpendicular volume projections from a simulated screw-sample. The simulation also contains a plate and two holes. In order not to obscure the summed projections, the plate is not shown in the 3D model. This figure is supposed to illustrate the transition from volumetric data, to 2D summed projections

The goal of this work is to train a convolutional neural network in a way that it becomes capable of inferring a specific rotation matrix from three input images. Applying the inferred rotation matrix then to each point (*x*, *y*, *z*) in a volume transforms a randomly oriented 3D object back to a previously defined and properly aligned position. The three images are the three orthogonal, summed volume projections along the x-, y-, and z-axis of a volume containing the joint (see Fig. 2). We stress the point that we are not talking about the original 2D X-ray projections from which the volume is reconstructed during or after a CT scan. The projections that were used as CNN input are CT volumes summed along a certain direction. Using three images instead of the entire volume as CNN input requires less computational resources, both for storing the data and processing it during the training. Due to the rivets’ rotational symmetry, using only one or two perspectives can produce ambiguous images. Furthermore, we assume that more than three perspectives do not produce any significant added value.

The original volumes were quite large considering the operations that needed to be performed on them in order to receive the data set (see Sect. 2.1.4). Their dimensions ranged from more than $500\times 500\times 500$ up to $1500\times 1500\times 1500$ voxels, which, in the 16bit case translates to approximately 250 MB and up to 6.75 GB, respectively. In order to reduce computation times during both dataset generation and training the volumes were therefore reduced to $256\times 256\times 256$ voxels. This reduction, however, limits the information value. The results presented in Sect. 3 therefore only apply to CT volumes of comparable dimensions and not CT volumes in general.

Instead of choosing the rotation matrix as CNN output, one could also use *Euler* angles, *Rodrigues* parameters, or quaternions. A comprehensive mathematical excursion into the field of rotations and affine transformations is beyond the scope of this work. However, we will concisely motivate in the following why we chose the rotation matrix as our network output.

Each rotation approach comes with intrinsic advantages and drawbacks. *Euler* angles are easy to comprehend, yet they can be ambiguous and the loss function of the neural network would need to be able to understand the periodic equality of angles (e.g., rotating by $42^{\circ }$ yields the same result as rotating by $402^{\circ }$). This applies partly to *Rodrigues* parameters (see Eq. 1) as well, since one of the four numbers is an angle. Another inconvenience regarding *Rodrigues* parameters is the loss function, which would have to be custom made. Using standard losses such as the mean absolute error (MAE) or mean-squared error (MSE) is therefore not possible. It has to consist of two parts, one for the angle-related loss and one for the vector-related loss. The interpretation of custom loss function outputs with contributions from different mathematical sets (angles and numbers with an absolute value $\le 1$) is non-trivial. From a machine learning point of view, a compound loss function therefore complicates the correct accounting of losses. It also jeopardizes the proper functioning of the network since a loss function needs to be differentiable in order to perform gradient descent. While *Rodrigues* parameters were not employed as network output for reasons mentioned above, they can be used for generating a well-curated data set, consisting of rotation matrices and image triplets, as we will show in Sect. 2.1. Like *Rodrigues* parameters, quaternions consist of two parts, a scalar (real) part which defines the amount of rotation, and a vector (imaginary) part, which defines the axis of rotation. This would lead to similar loss function-related inconveniences, as was the case with *Rodrigues* parameters. Additionally, quaternions have an abstract and complex (imaginary numbers) nature, which complicates their handling and interpretation from a machine learning point of view. The rotation matrix has the clear advantage of needing only one loss function, since all nine matrix entries are of the same nature, namely real numbers in the range of $\left[ -1, 1\right]$. In addition to that, this approach does not deal with angles and their ambiguities. The rotation matrix can, however, be calculated from *Euler* angles and *Rodrigues* parameters as well as from quaternions. However, the rotation matrix has the drawback of not only being able to rotate an object, but also to warp and to translate it if the matrix entries deviate from the intended pure rotation values. In the following, we investigate the magnitude of the warping effects produced by our own trained ResNet50 and if they are sufficiently small for the subsequent dimensioning task.

### Data generation and preparation

In the field of machine learning, it is important to use well-curated data. This ensures that the network does not develop a ”false” sensitivity for features that are irrelevant for the task at hand. In this context, computer scientists often quote the aphorism ”Garbage in, garbage out” [38], which dates back to an article from 1957 [39]. Therefore, we ensured that our data set was free of duplicates and ambiguous input target value pairs. The following paragraphs elaborate on our efforts to ensure a well-curated data set. Table 1 ﻿lists some of the adjustable CT-parameters that where used to acquire the respective volumes.

**Table 1:** Scan parameters and additional information relevant for acquiring the CT-volumes on which the algorithm was tested. Here, U: stands for the peak acceleration voltage, I: for tube current, FDD: for focus-detector-distance, FOD: for focus-object-distance, and M: for the magnification. Rivet A and Rivet B were acquired in the same scan, hence the identical parameters

	Scan parameters							System specifications	
	U	**I**	FDD	FOD	M	Filter	Voxelsize	Detector	System
	[kV]	[μA]	[mm]	[mm]	[–]	[mm]	[μm]	[–]	[–]
Rivet A	240	350	1100.00	315.53	3.49	Cu: 1.0	28.7	Waygate dynamic41|100	v|tome|x L 300D
Rivet B	240	350	1100.00	315.53	3.49	Cu: 1.0	28.7	Waygate dynamic41|100	v|tome|x L 300D
Rivet C	200	200	815.84	82.65	9.87	Cu: 1.0	59.4	Waygate dynamic41|100	v|tome|x L 300D
Rivet D	210	110	992.47	99.25	10.00	Sn: 0.5	113.8	Waygate dynamic41|100	v|tome|x L 300D
Rivet E	230	130	816.55	128.36	6.36	Cu: 1.5	31.4	Waygate DXR250	v|tome|x M 240D

#### Rodrigues

In order to have a better intuition of the effect of rotations around an arbitrary rotational axis, we chose to perform them with *Rodrigues* [44] parameters instead of *Euler* angles or quaternions. *Rodrigues* parameters consist of a unit vector ${\textbf {k}}=\left[ k_{\text {x}}, k_{\text {y}}, k_{\text {z}}\right] ^{\text {T}}$, around which the rotation of the sample occurs and an angle $\alpha$ that determines the magnitude of the rotation. Axis ${\textbf {k}}$ and angle $\alpha$ can then be inserted in the *Rodrigues rotation formula*, which in turn produces a rotation matrix ${\textbf {R}}$:

1 $$\begin{aligned} {\textbf {R}} = {\textbf {I}}+\left( \sin \alpha \right) {\textbf {K}}+\left( 1-\cos \alpha \right) {\textbf {K}}^{2}, \end{aligned}$$

with ${\textbf {K}}$ being the cross-product matrix of ${\textbf {k}}$.

#### Halton sequence

Randomly distributed vectors come with the danger of creating hotspots in their respective mathematical space and/or dimension. That way a distribution bias could be created, which possibly degrades the network’s performance. Therefore, we chose the *Halton* sequence [42] for the generation of vector-angle pairs. *Halton* sequences are quasi-random and distribute numbers more evenly. The procedure for generating vector-angle pairs is as follows:

1. A 4-dimensional *Halton* sequence is created with MATLAB’s *haltonset(d)* function, with $d=4$. The first three dimensions serve as the basis for the three spatial dimensions of the rotational vector ${\textbf {k}}$, and the fourth dimension serves as the basis for the corresponding angle $\alpha$.
2. For the *Rodrigues* rotation formula (Eq. 1) to work properly, the rotational vector ${\textbf {k}}$ needs to be normalized.
3. In order to limit the amount of angles and to ensure that the network is able to learn enough^5^ from every rotation, first, 64 angles are randomly selected from the fourth *Halton* dimension. They serve as our fundamental set of angles. Then, for every rotational vector ${\textbf {k}}$, 16 of the 64 angles are selected via random permutation.
4. Only positive angles where permitted.

#### Final clean-Up

Based on the rotational symmetry of the used self-piercing rivets and the mathematics of rotations, there are two major and one minor data-related pitfalls to avoid. The two major pitfalls correspond to rotations that have different rotational parameters yet yield the same outcome:

1. If the rotational axis ${\textbf {k}}$ is parallel or anti-parallel to the rivets symmetry axis ${\textbf {k}}_{\text {sym}}$, every rotation around that axis will produce more or less the same outcome.^6^
2. If $\alpha =0$, the outcome is independent of ${\textbf {k}}$ and the results will look identical for every ${\textbf {k}}$
  Very small deviations from both (1) and (2) could potentially confuse the network. Therefore, rotations with a cross-product ${\mathbf{k}} \times {\mathbf{k}}_{{{\text{sym}}}} = 0 \text { or} \approx 0$ and rotations with $\alpha =0$ were removed from the data set.
  Lastly, (3) all rotational outcomes were compared with each other based on *MATLAB’s* structural similarity index function ssim(...) [12]. Here, indices $\approx 1$ indicate greater similarity than indices $\approx 0$. If the projections of one rotation were too similar (ssim-index $\ge 0.9$) to the projections of another rotation the former rotation was automatically removed from the data set. The resulting $\left( {\textbf {k}}, \alpha \right)$-pairs produce the rotation matrices **R** that represent the target values to which the CNN is supposed to map the input data (volume projections). As a result, we created a data set with 7269 unique and sufficiently different rotations. These rotations served as the foundation for the generation of the volume projection data set.

#### Generation of projections

Every $\left( {\textbf {k}}, \alpha \right)$-pair of the cleaned data set was then used to rotate a $256\times 256\times 256$ reconstructed volume of a self-piercing rivet. The rotated volume, which has the same dimensions as the pre-rotation volume, was then summed up along its x-, y-, and z-axis in order to create the three input projections for the CNN. In total, five data sets were generated, based on five different rivets (A–E). The composition and respective size of the data sets can be seen in Table 2. Every data set mix was split into a training, validation, and test set. The amount of every rivet in the largest data set (Mix4), which was later used for training, can be seen in Table 3. Exemplary projections of the rivets after rotation along with their corresponding $\left( {\textbf {k}}, \alpha \right)$-pair and rotation matrix ${\textbf {R}}$ can be seen in Table 5. The final result of this generation process is (1) a CSV-file that stores the projection file names and the respective rotation matrix as well as the *Rodrigues* rotation parameters, and (2) a folder with all the projections in TIFF format.

In addition to splitting Mix4 into a training, validation and test set, we performed the hold-out method for each rivet. Table 4 shows the rivet distribution in the case of holding out Rivet C as the test set. By doing so possible outliers can be identified. Furthermore, the networks ability to generalize to new data instances can be investigated.

**Table 2:** Data sets and their number of both unique and total rotations

Mix	Content (rivets)	Unique	Total
		Rotations	Rotations
0	1 rivet: A	7269	7269
1	2 rivets: A+B	7269	14538
2	3 rivets: A+B+C	7269	21807
3	4 rivets: A+B+C+D	7269	29076
4	5 rivets: A+B+C+D+E	7269	36345

**Table 3:** Final data set (Mix4) and the amount of each rivet in the training, validation, and test set, respectively. The distribution was achieved with a random shuffle of the entire data set

Rivet	Instances	Instances	Instances
	In training set	In validation set	In test set
A	5784 (19.9%)	761 (20.9%)	724 (19.9%)
B	5815 (20.0%)	737 (20.3%)	717 (19.7%)
C	5818 (20.0%)	723 (19.9%)	728 (20.0%)
D	5790 (19.9%)	693 (20.1%)	786 (21.6%)
E	5869 (20.2%)	720 (19.8%)	680 (18.7%)
Total	29076	3634	3635

**Table 4:** Exemplary rivet distribution when one rivet (here it is Rivet C) is used as a hold-out set. Experiments with Mix4 suggested that approximately 16×10^3^ samples should be enough for the contextual task and data sets

Rivet	Instances	Instances	Instances
	In training set	In validation set	In test set
A	2487 (24.2%)	623 (6.1%)	0 (0%)
B	2604 (25.3%)	646 (6.3%)	0 (0%)
C	0 (0.0%)	0 (0.0%)	2573 (100.0%)
D	2581 (25.4%)	632 (6.5%)	0 (0%)
E	2618 (25.1%)	672 (6.1%)	0 (0%)
Total	10290	2573	2573

**Table 5:**
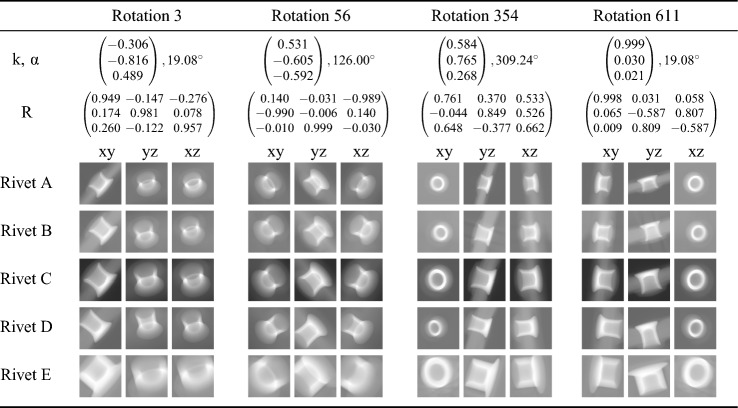
Exemplary rotations from the data set. Images xy, yz, and xz serve as input to a convolutional neural network and the rotation matrices R serve as the labels. The Rodrigues parameters k and a are the foundation for generating a uniformly distributed data set of rotations. They were used to generate the rotation matrices. Angles were rounded to two decimal places. The influence of this rounding on the angle is assumed to be negligible. Matrix- and vector-related quantities, however, were rounded to three decimal places. This is for ensuring the possibility of quantifying the error when evaluating the network’s performance

### Mapping projections to 3D rotation matrices

The core idea of machine learning is, in the context of supervised learning and artificial neural networks (ANN), to find a mathematical function (the network consisting of its weights and biases) that accurately maps one set (the input and its features) to another set (the output labels).

**Fig. 3:**
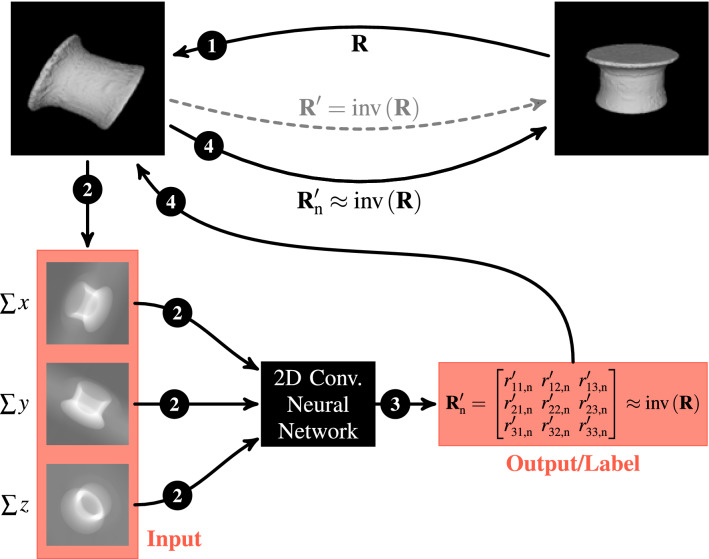
Workflow of our approach: The goal is to find a 2D CNN that maps three images to their corresponding rotation matrix with sufficient accuracy. The numbers in the solid black circles indicate the order: (1) The volume is rotated with a rotation matrix ${\textbf {R}}$. (2) The three summed volume projections from the randomly oriented rivet are passed to the network. (3) The network predicts a rotation matrix based on the three projections. (4) The predicted rotation matrix is used to rotate the volume back to its original orientation. ${\textbf {R}}'$ is the inverse rotation matrix or, respectively, the rotation matrix from our data set, which the network is supposed to learn. ${\textbf {R}}'_{\text {n}}$ is the resulting rotation matrix from the network

The aim of our approach is to train a 2D convolutional neural network (CNN) with a large and diverse enough data set until it is able to accurately map three 2D (summed) projections to a corresponding 3D rotation matrix (see the workflow in Fig. 3).

While training a 2D regression CNN on such a data set works quite well from a machine learning point of view (as we will show in Sect. 3), using the 3D rotation matrix as the objective has two drawbacks: The network has to minimize the loss function for a total of nine numbers, and, much more importantly, the tolerable deviation is very small due to the spatial transformation ability of 3D matrices: Next to rotations, matrices can also be used to warp and translate 3D objects. Therefore, we also investigated the magnitude of undesired deformations and/or translations, which we will also show in Sect. 3.

### Network architecture

We chose to slightly modify the widely used ResNet50 [19] architecture in a way that supported our endeavor, meaning the original soft-max output layer, generally used for classification, was cut off and replaced with a few dense layers. This modification enables the network to be used for learning regression tasks instead of classifying objects. It has been exhaustively researched and shown that ResNets can be readily modified and used for regression tasks (see, for example, [11]). In Keras it is possible to employ a ResNet50 with pre-trained weights from the ImageNet data set[9], which consists of more than $14\times 10^{6}$ RGB-images and more than $20\times 10^3$ classes. We therefore had to consider whether or not we incorporated the concept of *transfer learning* and used the pre-trained version of Keras’ ResNet50. There are two main differences between our data set and ImageNet, which need to be considered: 1) ImageNet data instances consists of RGB images with three channels, while our data instances consist of three grayscale images with one channel each. There is a study [18] that suggests that color does not seem to be a critical feature for learning. We did not investigate if this conclusion can be transferred to our data set, since our images are naturally grayscale or single channeled, respectively. 2) RGB images maintain orientations across channels, meaning that a horizontal line, for example, is horizontal in all three channels. This is not the case in our data set, where the three channels show different perspectives of a single object, thereby destroying the cross-channel homogeneity. Because of all these factors, we decided to train our ResNet50 model from scratch and therefore not to apply transfer learning.

A quick hyperparameter search was performed on the layers of the newly added regression block in order to identify a combination that improves the network’s performance. In order to speed up this process, several network settings were trained, evaluated, and compared with only 8000 randomly chosen examples from Mix4 (see Table 2). The hyperparameters investigated were as follows:

1. Number of dense layers $N_{\text {d}}$ in the new block^7^ with $N_{\text {d}}\in \left[ 1,...,4\right]$,
2. number of units in each dense layer $N_{\text {u}}$ in the new block,^8^ with $N_{\text {u}} \in \left[ 3600, 1200, 900, 360, 120, 54, 27, 9\right]$,
3. dropout layers between the dense layers and their dropout rate $r_{\text {d}}$, with $r_{\text {d}} \in \left[ 0.2, 0.5, 0.7\right]$.

In addition to this, all activation functions in the base model were replaced by *tanh*-activation functions. This is due to the fact that the target values, which are the nine matrix entries, are in the range $\left[ -1,.., 1\right]$, which is also the codomain of the *tanh*-function.

Considering only these variations, the best performing network for mapping three summed projection images of a randomly rotated rivet to the corresponding rotation matrix, and for rotating it back to its previously defined position (see Fig. 3), had the following architecture and parameters:

$$\begin{aligned} \text {RN50}_{\text {base}} \mapsto \text {GAPL} \mapsto \text {DL}\left( N_{\text {u}}=900\right) \mapsto \text {DO}\left( r_{\text {d}}=0.5\right) \mapsto \text {DL}\left( N_{\text {u}}=54\right) \mapsto \text {Output}, \end{aligned}$$

with $\text {RN50}_{\text {base}}$ being the base of a conventional ResNet50 without pre-trained weights and without the last *softmax*-layer, GAPL being a 2D global-average-pooling layer, DO being a dropout layer, and DL being a dense layer.

### Size of the data set

All data sets (Mix0 to Mix4 in Table 2) presented in Sect. 2.1.4 were compared against each other. More concretely speaking, the CNN with a ResNet50 base, presented in Sect. 2.3, was trained with varying amounts of each data set. Every individual data set $\text {S}_{i}, i\in \left[ 0,1,2,3,4\right]$ was split into 80 batches $\text {B}_{j}$ with $j\in [0,1,2,...{79}]$. Furthermore, $\text {S}_{i,k} = {\bigcup _{j=0}^{k}\text {B}_j}$ and $k\in [0,1,2,...{79}]$. So, for example, $\text {S}_{3,56}$ is a data set, consisting of 56 batches of data set $\text {S}_{3}$ (rivets A, B, C, and D). The network was then trained with increasing amounts of data of each set, as described in Algorithm 1:
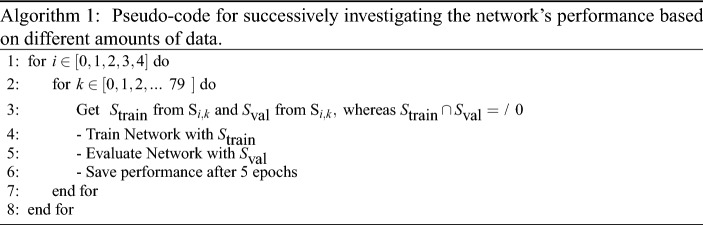


This technique allows quantifying the variety of the data set and the actually needed amount of training data. A very diverse data set will probably require the entire data set for getting a sufficiently well-performing network. In our case, however, it might suffice to only use a subset of the entire data set (as we will show in Sect. 3). This approach can speed up training, since less data is needed but the network will still ”see” all relevant data.

## Results

**Fig. 4:**
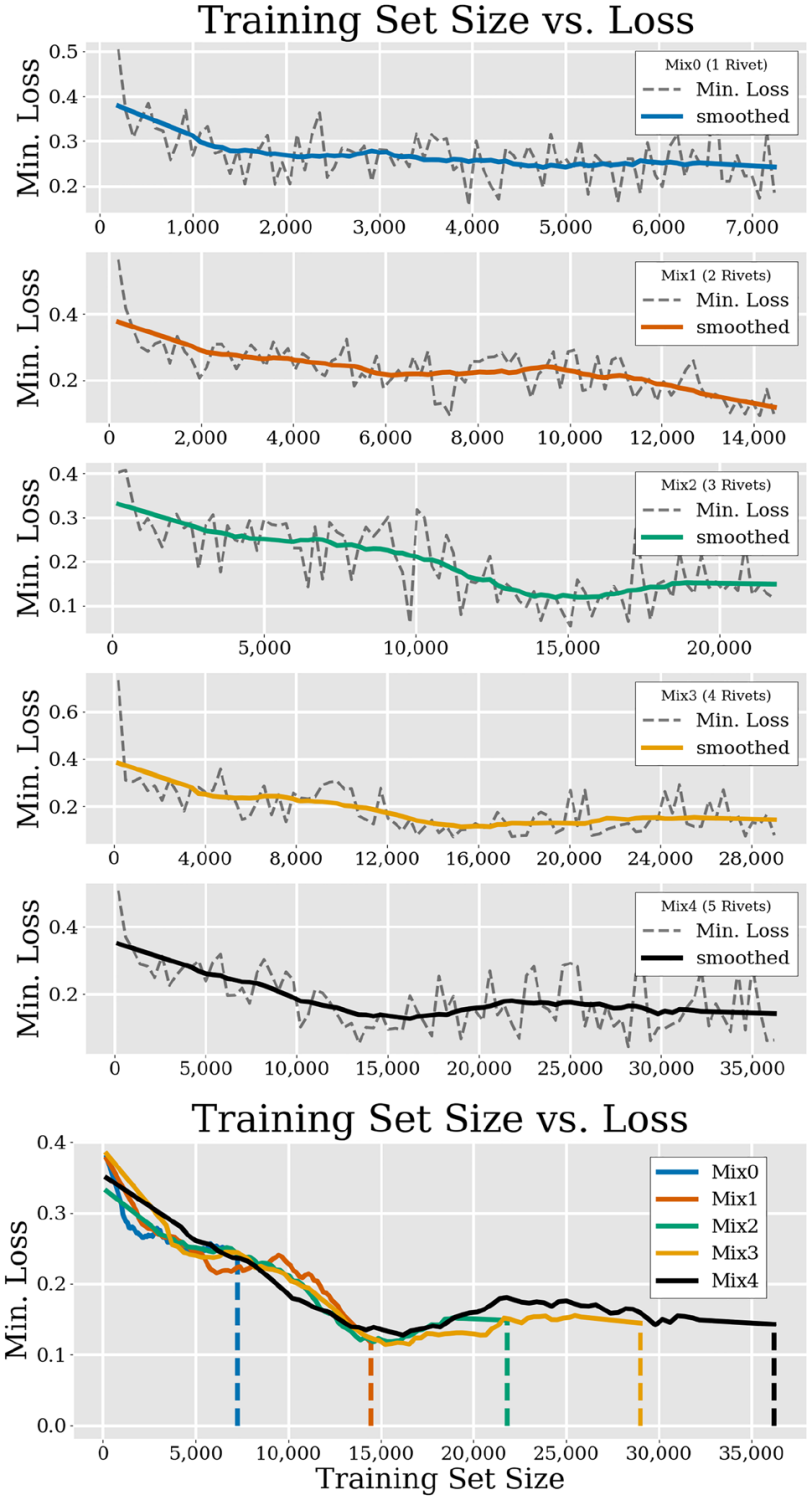
First five graphs: Individual data sets and their performance with each data set size after five epochs. Bottom graph: All runs superimposed. The dashed lines indicate the entire data set size of the respective data set

Figure 4 shows how the individual training sessions described in Sect. 2.4 performed depending on the amount of data from each set. In the bottom graph of Fig. 4, it can clearly be seen that the performance first improved with increasing amounts of data until it reached a set size of approximately $16\times 10^3$ examples in Mix2. After that point, the performance seems to level out.

**Fig. 5:**
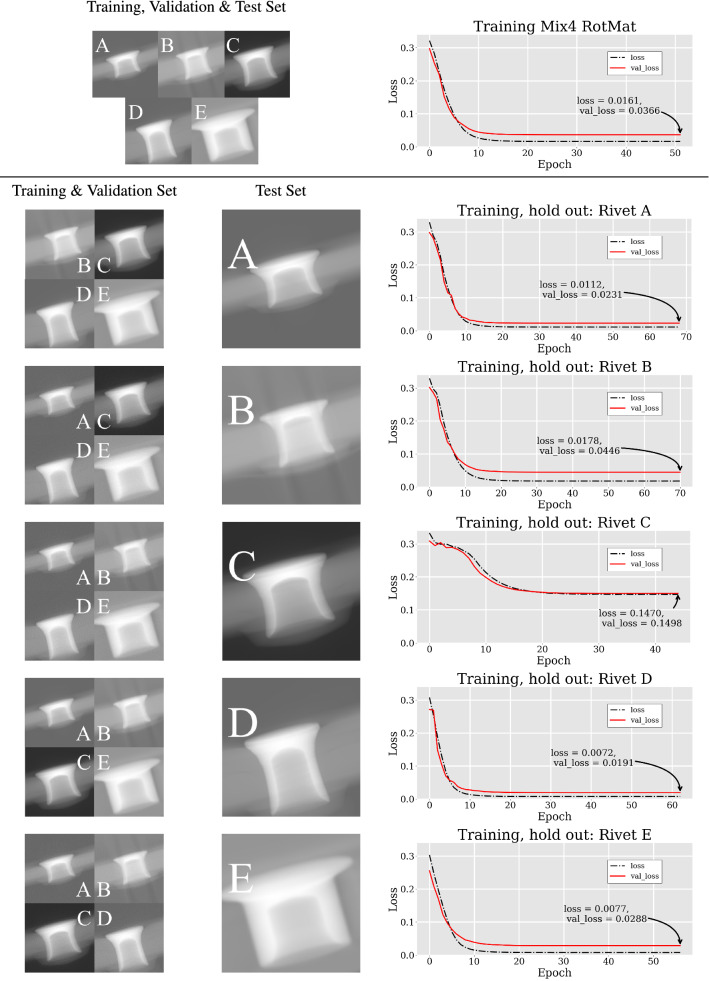
Top: Training with the entire Mix4 set. Bottom: Training with hold-out sets. Here, in each training one rivet was removed from the training and validation set and was used as a test set (hold-out set). Using Rivet C as the hold-out set for testing had the largest impact on the training if compared with the other hold-out runs

Figure 5 shows the training and validation performance of our modified ResNet50 for both the approach with Mix4 (top) and the approach where one rivet was used as a hold-out set (bottom). The training process was individually interrupted because we set ${EarlyStopping}=15$ epochs.

**Fig. 6:**
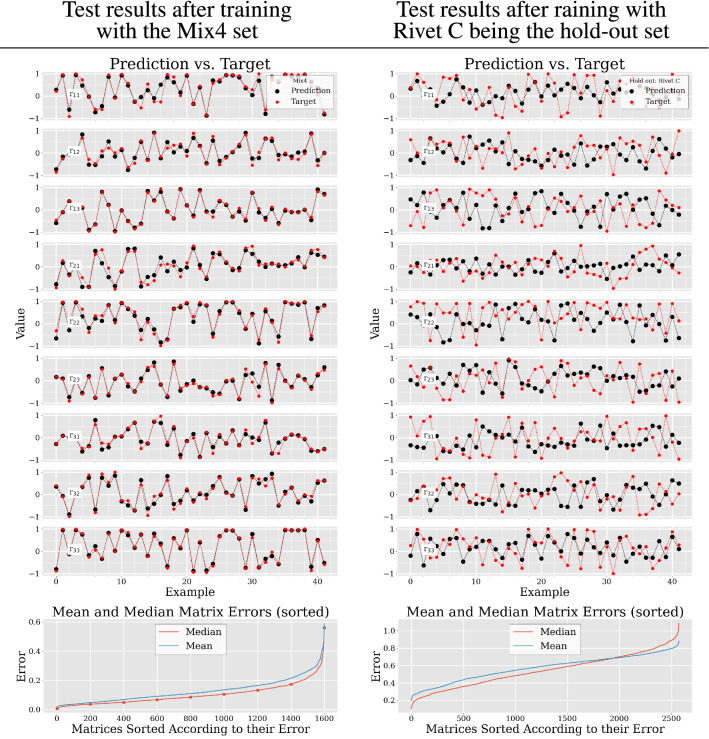
All graphs on the left refer to the Mix4 data set. All graphs on the right refer to the training and testing with Rivet C being the hold-out set. Top graph: Training loss and validation loss of our modified ResNet50. Center graphs: Exemplary predictions versus target values of the test data set. The closer a red dot (label) is to its respective black counter part dot (prediction) the smaller the error in this particular case. The dotted lines between the points were added to make the respective pairing of prediction and target clearer. Bottom graph: Matrix mean and median errors in sorted order. Here, all matrices are sorted according to their mean and median errors in ascending order. This shows, that in the case of the Mix4 data set the majority (approximately 80%) of the matrix errors stay below 0.2. However, when Rivet C was the hold out set, almost every predicted matrix produced errors larger than 0.2. The red dots in the graph on the bottom left mark the examples that are shown in Fig. 9

Figure 6 depicts the similarity of prediction versus target value. The left side shows the results after training with the Mix4 set and the right side shows the results after training without Rivet C, but instead using it as the hold-out set, or test set, respectively. The nine scatter graphs on each side show 42 examples from every individual matrix entry $\text {r}_{11} \text { to } \text {r}_{33}$ and how well the prediction of each entry matches with its target value. These values were produced by inputting examples from the respective test set to the trained network. Values with the same x-axis value (”Example”) belong to a single rotation matrix. The two smooth graphs on the bottom of Fig. 6 show the test set’s mean and median error of each matrix sorted in ascending order.

**Fig. 7:**
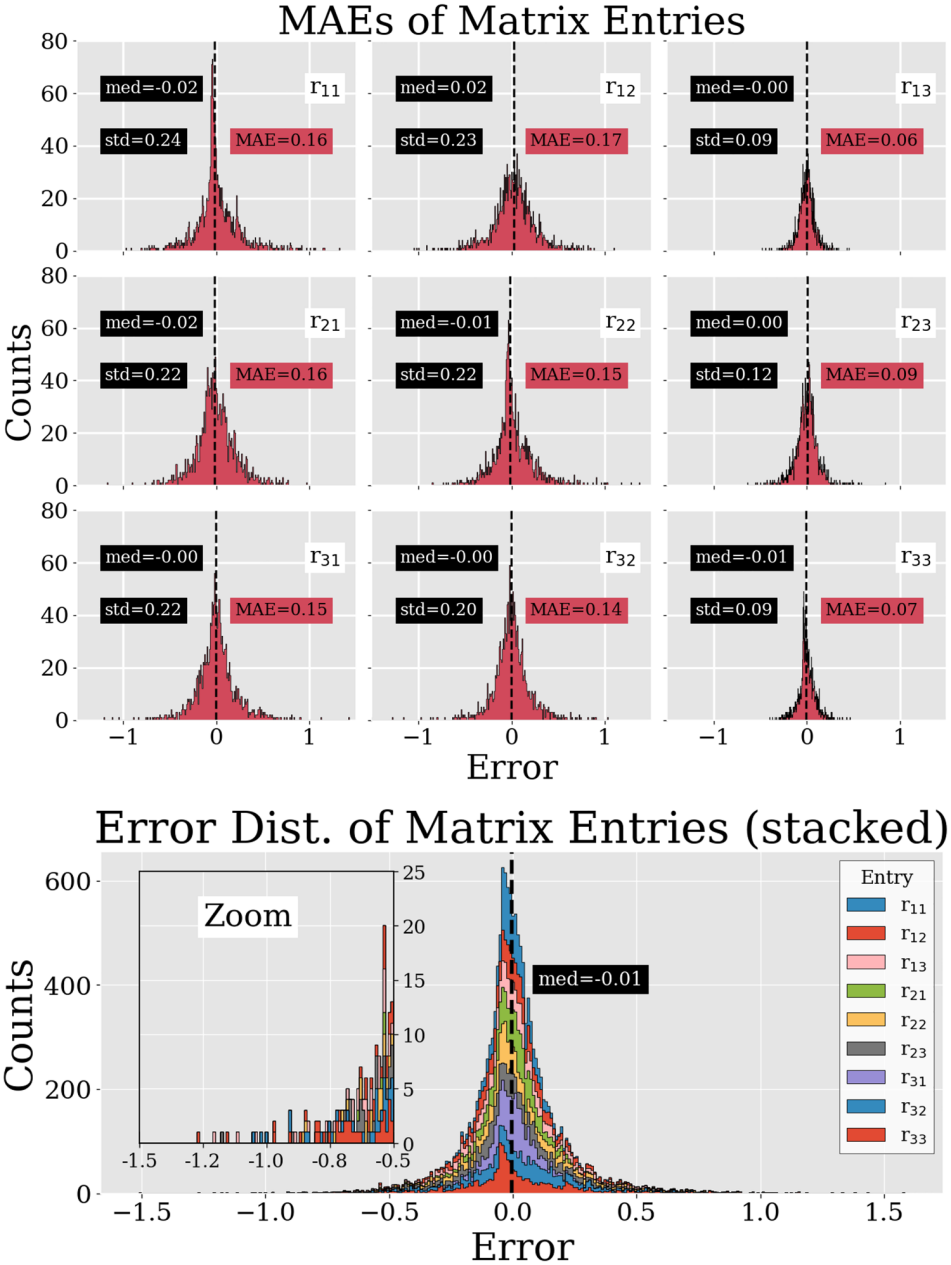
Top graph: Error distribution of all matrix entries with entry-specific (${r_{11}\text {--}r_{33}}$) mean absolute error (MAE), median (med), and standard deviation (std). Bottom graph: Stacked overlay of the individual error distributions (top nine graphs) with an enlarged section in order to conceptualize the idea of the amount of larger errors. Both top and bottom graphs are based on the Mix4 test set

The top part of Fig. 7 shows the error distribution of all matrix entries with the entry-specific mean absolute error (MAE) and median, which is more robust against outliers than the mean. For this the Mix4 set was used. The lower part of Fig. 7 shows all distributions in one graph.

**Fig. 8:**
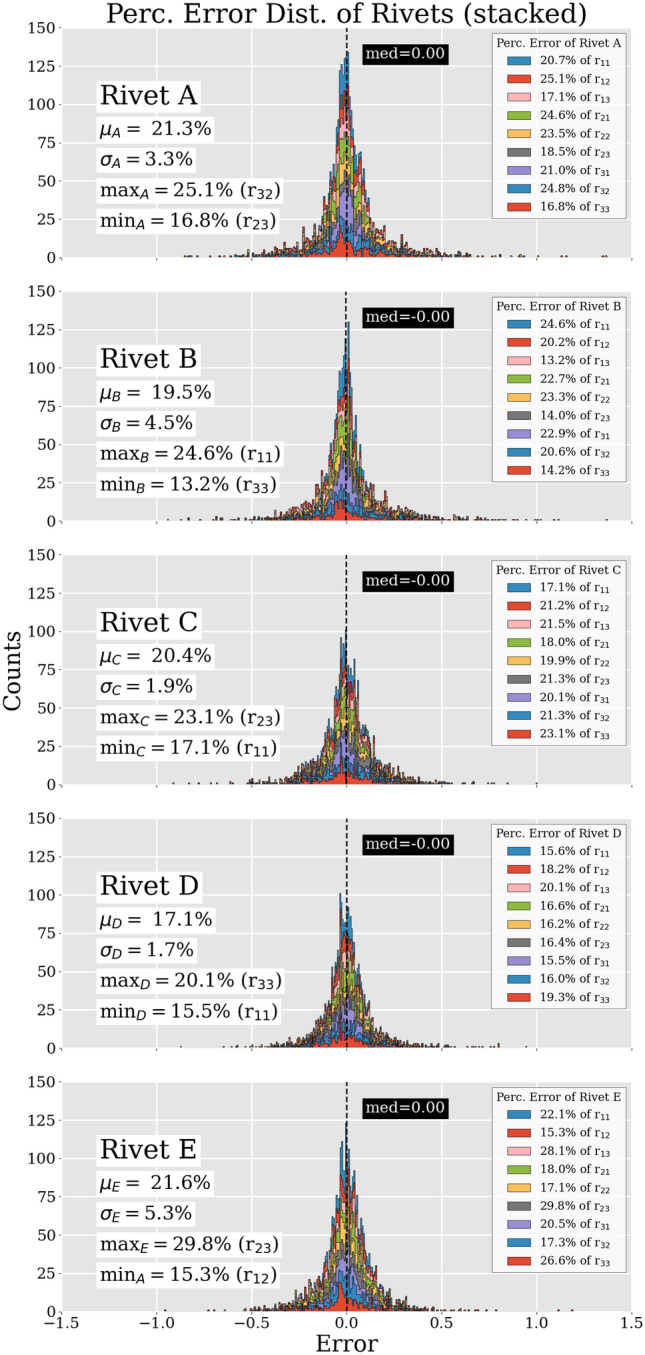
Percentage error of rivets (Mix4 test data set) of the respective matrix entries. Here we see both the mean contribution of each rivet to the entire error ($\mu$, $\sigma$) and their contribution to each matrix entry (${r_{11}\text {--}r_{33}}$) including their respective maximum and minimum contribution (max, min)

Figure 8 shows in detail how much the rivets contributed individually to the total error of every matrix entry. Here also, the Mix4 set was used. In addition, the respective means (e.g., $\mu$), standard deviations (e.g., $\sigma$) as well as minimum and maximum of every rivet with respect to their percentage errors of the matrix entries are shown.

**Fig. 9:**
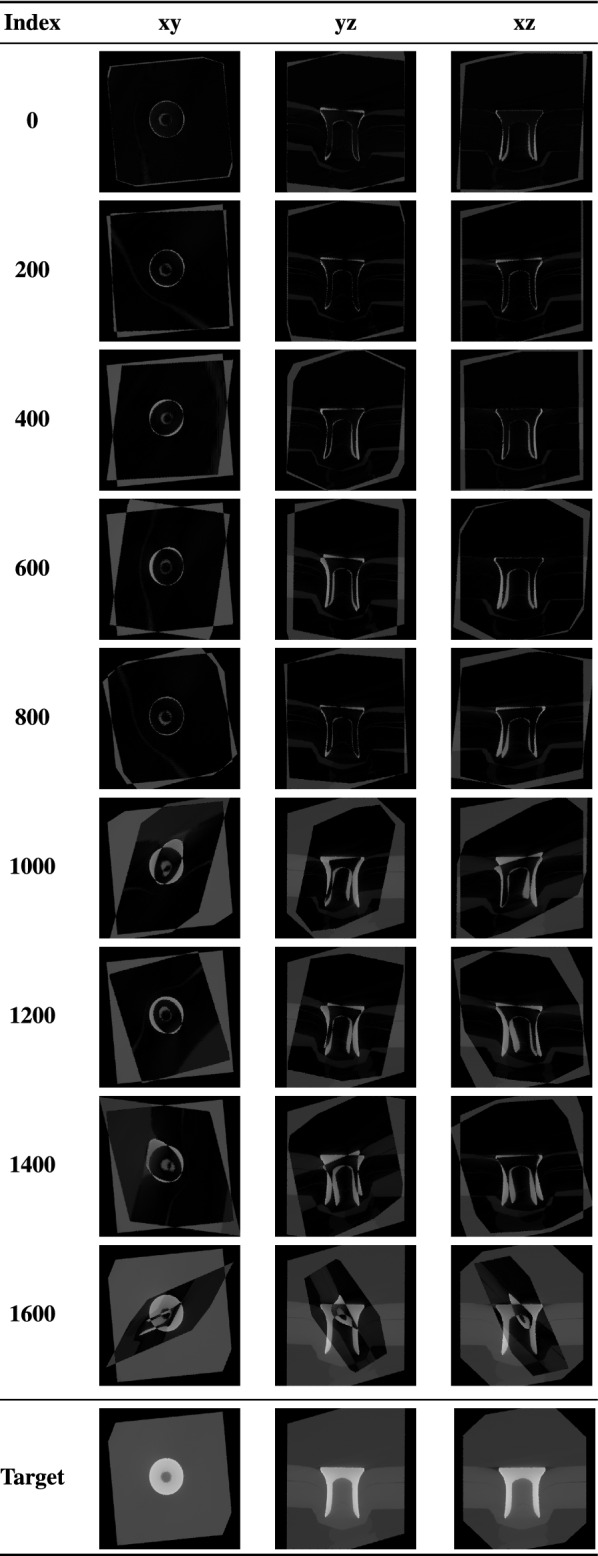
Exemplary visualization of the severity of deviations from pure rotation matrices and the resulting warping effects. The indices correspond to the x-axis of the bottom left graph in Fig. 6, which shows the mean and median errors of the predicted matrices in ascending order. The higher the index, the larger the deviation is. Here the Mix4 set was used as well. The difference between the target rotation and the prediction rotation is depicted. In other words, every image line shows three cutting planes (xy, yz, xz) of the subtraction of two volumes (target volume and volume after rotation with predicted matrix). If the prediction rotation resulted in a volume that is identical to the target volume (zero error), the volume subtraction would produce completely black images. Dark pixel values correspond to small deviations whereas bright ones correspond to large deviations. The first two rows (Index 0 & 200) show predominantly dark pixels, which indicates, that the prediction and the objective in the last row where almost identical

In Fig. 9 some examples of the predictions from the Mix4 test set are shown. In order to visualize the deviations from the target rotation we did the following: A rivet was rotated with a rotation matrix from the Mix4 test set. This resulted in a randomly oriented volume. In a next step, the predicted rotation matrix was applied to the randomly oriented volume, which aimed at bringing the volume back to its original, aligned position (see last row in Fig. 9). By subtracting the resulting volume from the original one, we can visualize how much they differ in a qualitative fashion. This was done only for the Mix4 test set, since the errors of the hold-out approach are too large.

## Discussion

As was mentioned in the last part of the Sect. 1, we investigated two scenarios. The first being that a neural network that is subjected to rather constant rivet demographics. This scenario represents a constant riveting process as well as CT scans with a constant set of parameters. In the second scenario a neural network is subjected to new data with unknown rivet demographics. This scenario represents changes in either the riveting or the CT imaging process. These changes can concern new materials, geometries or CT parameters.

The presented results and argumentation apply only to CT volumes that possess similar characteristic features and dimensions as the five CT volumes with rivets presented in Sect. 2.1.4. In order to successfully apply our methodology to volumes with new rivets that are different from the ones in Sect. 2.1.4, more samples need to be manufactured and new data sets need to be generated. This includes different rivet geometries and varying scan parameters on the CT system like voltage, current, or magnification.

The results in Fig. 4 suggest that the variety of the five respective data set mixes can largely be learned with only $16\times 10^3$ examples, provided they are generated as was described in Sect. 2.1. This follows from the fact that as the number of different rivets *i* increased, each of the data set mixes $\text {M}_i$ consisted of increasing amounts of different rivets and therefore possessed a greater variety for which the network had to find a more general mapping, therefore avoiding the danger of overfitting on a single rivet.

From a machine learning point of view the results in Fig. 5 are promising. The loss functions in the graphs on the right subsequently and steadily converge to a low value. This shows that the network is continuously learning. The network’s performance only seems to become weaker in the case of using Rivet C as a hold-out set. Comparing the different hold-out scenarios, one could assume that the difference in grey values is the reason for the poor performance. This discrepancy is most striking when considering Fig. 6. Here the test set performance is quite different. In the case of using the Mix4 set the error of predicted matrices is relatively small when compared to using Rivet C as a hold-out set. These results suggest, that not the different geometries of the rivets but their grey value distribution has a large impact on the networks performance. Yet, the nature of the rotation matrix dictates that deviations from the intended matrix values, could, aside from rotations, also result in scaling or shearing of a volume. The question now is whether or not the magnitude of deviations as shown on the left side (Mix4 set) in Fig. 6 can be neglected in that context.

A quantitative analysis of the results, produced with the test data set from Mix4, can be seen in Fig. 7 and in Fig. 8. In the former graph, the error distributions of the individual matrix entries are shown. Their individual distributions seem to be without any noteworthy bias besides an almost imperceptible collective shift toward negative values. This can be seen in the lower part of the graph, in which all distributions were stacked on top of each other. The individual distributions from the top part show that the standard deviation from r$_{13}$, r$_{23}$, and r$_{33}$ are about twice as small as from the other distributions. This means that the network was able to predict the correct values of r$_{13}$, r$_{23}$, and r$_{33}$ more accurately than the others.

In order to get an idea of how much every rivet contributed to the error of the individual matrix entries, we plotted the respective distributions in Fig. 8 and calculated the percentage error of every rivet. For this we used the Mix4 set again. In each legend, we list the percentage error of the respective rivets. So, for example, rivet A contributes to the total error of r$_{11}$ with 20.7%. By taking the mean of all percentages of a rivet, we end up with the mean error contribution (e.g., $\mu _{\text {A}}$) of that specific rivet to the data set. We initially prepared the data set with five different rivets. Every rivet should therefore contribute approximately one-fifth to the final error, otherwise the data set is not homogeneous enough. Or in other words, some rotations of a specific rivet differ more from other, more similar rivets. However, the rather small deviations are tolerable, since we randomly shuffled the data set and then only used a subset ($16\times 10^3$) of it. All in all, Fig. 8 shows that while the rivet volumes are clearly distinguishable from one another, the network’s performance was not influenced disproportionately by only one rivet in the case where we used the Mix4 set for training, validation and testing. This represents scenario one, which was mentioned in the beginning of this section.

The mean and median errors of every predicted rotation matrix was calculated and visualized in the two bottom graphs of Fig. 6. It shows that there are apparently outliers in every prediction. However, more interesting is the fact that in the bottom left graph (Mix4) most ($\approx 80 \%$) matrix-specific mean or median errors stay below 0.2. Still, just an error of 0.2 can cause undesired warping or translatory effects, as we will show in the following. The qualitative results of selected examples along the median curve of Fig. 6 are depicted in Fig. 9. Here, the severity of deformations, caused by small deviations from the target rotation matrix, are shown graphically. The last row (index 1600) in particular shows the warping effects we mentioned earlier. The result of the rotation looks nothing like the target. The predicted rotation in the first row (index 0) of Fig. 9 was closest to its target. However, the slight tilt and the almost imperceptible warping are noticeable. While this does not invalidate our approach in general, it clearly invalidates the performance of our trained ResNet50, especially if after rotating the dimensioning of key features should be performed.

## Conclusion

The underlying application for our endeavor, is the automatic rotation of a randomly oriented rivet in a CT volume to a position from which key features can be easily dimensioned. We showed that our proposed method bears the potential of deducing a respective rotation matrix relatively accurately, based on three orthogonal volume projections of a misaligned mechanical joint in a CT volume. We not only present how the contextual data set should be generated but also that the CNN performs well on that data set from a machine learning point of view. This statement shows especially in the loss curves of Fig. 5 where both loss and validation loss drop quickly and converge around rather small values. The number of unique rotations, presented in Sect. 2.1.3 should be sufficient and does not need to be increased, provided that the demographic of the data set does not change. This follows on one hand from our argumentation in Sects. 2.1.2 and 2.1.3. In these sections we elaborate on making sure that the (**k**, $\alpha$)-pairs are distributed homogeneously and without duplicates or unwanted special cases that could confuse the network. On the other hand, Fig. 4 shows that more rotation-related data would not have increased the networks performance notably. Our hold-out approach (see Fig. 5), however, suggests that more data with different grey level distributions might improve the networks performance for the previously explained second scenario. In a regression task, such as the one presented here, small deviations from the desired target value are generally unavoidable and therefore oftentimes accepted since the performance of the network is by and large sufficient. The fundamental question of this work therefore was whether or not the rotation matrix is a suitable objective for a CNN to learn and if the magnitude of the deviations between target and prediction makes the result impracticable. We have shown that the performance of the network trained with our methodology is insufficient from the application point of view due to the large impact that individual deviations can have. This is due to the fact that deviations with magnitudes like they are presented in Sect. 3 can warp or translate the original volume to an unacceptable degree. We therefore suggest that while learning rotations from three orthogonal volume projections seems possible per se, an approach with a potential learning objective not prone to warping effects should be developed. The latter means one has to accept and deal with the inconveniences mentioned in Sect. 2.

## Outlook

There are, of course, many types and variations for mechanical joints. In order to perform well on more than just the type of rivets presented in this work, a more diverse data set needs to generated—physically as well as digitally. Additionally, the CT parameter space should be increased and varied. For example, parameters like voltage, current, magnification, or X-ray filters can be varied. Their impact on the resulting data quality, and therefore on the network performance, can then be analyzed in a similar way as was presented in our work. This would make the network more robust for the previously mentioned second scenario. Also, the hyperparameter search could be both extended and refined. Perhaps there is a more promising network architecture for the stated problem than the well-known and largely used ResNet50. One could also try to incorporate algebraic rules that only hold for pure rotation matrices, therefore forcing the network to reduce the effects of collateral warping. If, after all this, the rotation matrix still does not pose as a suitable target for the neural network, we suggest to adapt the presented network and incorporate a loss function that can handle *Rodrigues* parameters.

## Data Availability

The datasets generated during and/or analysed during the current study are available from the corresponding author on reasonable request. Physical samples as well as the used X-ray CT systems are property of the BMW AG, München.

## Footnotes

1 This is necessary to remove fine grinding residue and to decrease its reflectivity, which, if too high, would make microscopical inspection impossible.

2 It is believed that three factors caused this trend: (1) the large amounts of available data, (2) the amount of available computing capacity, and 3) the evolution of algorithmic techniques [17].

3 Usually via a method called gradient descent.

4 Possible changes are new rivet geometries or new parameters for the CT-scan.

5 The degree of new information presented to the network is greater between, for example, $5^{\circ }$ and $25^{\circ }$ than it is between $5^{\circ }$ and $5.5^{\circ }$.

6 Provided the rivet has no larger deformations or possesses defects that would break the rotational symmetry around that axis.

7 Excluding the output layer.

8 Except the output layer, which had nine units.
